# A novel mutation in the DYNC1H1 gene causing developmental and epileptic encephalopathy treated with ketogenic diet: A case report

**DOI:** 10.1097/MD.0000000000043277

**Published:** 2025-07-11

**Authors:** Fen Zhao, Lina Sun, Wandong Hu, Hongwei Zhang

**Affiliations:** a Department of Neurology, Children’s Hospital Affiliated to Shandong University, Jinan, China; b Functional Examination Room, Anqiu People’s Hospital, Weifang, China.

**Keywords:** case report, developmental and epileptic encephalopathy, *DYNC1H1* gene, ketogenic diet

## Abstract

**Rationale:**

DYNC1H1 variants are associated with a spectrum of neurodevelopmental disorders, such as spinal muscular atrophy, severe intellectual disability, and epileptic encephalopathies, with the majority of observed cases attributed to de novo variants.

**Patient concern:**

A 1-year-old Chinese boy presented with frequent seizures and developmental delay.

**Diagnoses:**

Cranial magnetic resonance imaging revealed malformations of cortical development. EEG indicated epileptic spasms and focal to bilateral tonic-clonic seizures. Trio-WES identified a *de novo* missense variant (c.3371A > G) located in exon 14 of the *DYNC1H1* gene, which was confirmed by Sanger sequence. The final diagnoses were “*DYNC1H1*-related developmental and epileptic encephalopathy; malformations of cortical development.”

**Intervention:**

Initial treatment with various ASMs proved ineffective. Finally, ketogenic diet treatment was introduced.

**Outcomes:**

The patient had achieved significant seizure control, and the follow-up EEG discharges were reduced.

**Lessons:**

This report expanded the genotypic spectrum of *DYNC1H*1 gene, and highlights the potential therapeutic option of ketogenic diet for *DYNC1H1*-related developmental and epileptic encephalopathy, particularly in cases refractory to ASMs. These findings contribute valuable insights for the precision medicine approach in treating such patients.

## 1. Introduction

*DYNC1H1* gene (OMIM: 600112) encodes dynein cytoplasmic 1 heavy chain 1 (DYNC1H1), a critical component of the cytoplasmic dynein motor complex functioning. This complex functions as a microtubule-associated protein and is essential for mediating retrograde axonal transport and other vital intracellular processes.^[1,2]^ Located on chromosome 14q32.31, the gene comprises 78 exons and encodes a large protein of 4646 amino acids.^[3]^ DYNC1H1 is highly conserved evolutionarily and exhibits widespread expression across multiple tissues, particularly in the brain, underscoring its importance in neuronal function.^[1]^

*DYNC1H1* variants have been implicated in a spectrum of neuromuscular and/or neurodevelopmental diseases, including hereditary spastic paraplegia, spinal muscular atrophy, severe intellectual disability, malformations of cortical development (MCD), or developmental and epileptic encephalopathy (DEE).^[4]^ Previous studies have demonstrated that heterozygous variants in the *DYNC1H1* gene are associated with abnormal brain morphologies as well as motor and sensory neuronal deficits.^[5]^ Notably, variants in different domains or different locations of the same domain of *DYNC1H1* gene tend to manifest distinct clinical phenotypes, highlighting the genetic heterogeneity of this gene.^[2,6]^ Consequently, the occurrence of novel DYNC1H1 variants is crucial for elucidating the underlying mechanism of the link between genotypes and phenotypes. Furthermore, a significant proportion of children harboring *DYNC1H1* mutations present with MCD and drug-resistant epilepsy.^[7]^ Thus, the search of the effective therapeutic strategies is necessary for patient with *DYNC1H1*-related epilepsy.

In this study, we presented a case of a child with DEE caused by novel heterozygous missense variant in the *DYNC1H1* gene. Notably, the patient demonstrated significant clinical improvement following treatment with a ketogenic diet (KD). This report highlights the potential therapeutic efficacy of KD in managing *DYNC1H1*-related DEE and may provide clinical reference for diagnosis and treatment of this condition.

## 2. Case presentation

### 2.1. Clinical history

In March 2023, a 1-year-old Chinese male infant was admitted to our hospital due to frequent seizures and developmental delay. Since birth, he had presented gradual developmental delay. At the age of 1 year, he started experiencing 2 types of seizures. One was characterized by limb stiffness, unresponsiveness, staring, and cyanosis, without accompanying limb tremors or drooling. Each episode lasted approximately 20 seconds and occurred about twice daily. Another was characterized by nodding or cessation of movement occurring in clusters, with each cluster lasting 10 or more times and lasting for several seconds to several minutes. The episodes occurred up to 7 to 8 times per day. He was the second child born to a non-consanguineous couple, with both parents and his older brother being healthy. His mother was conceived naturally, and both pregnancy and delivery were uncomplicated. His birth weight was 3.2 kg, and there was no history of perinatal asphyxia.

**Figure 1.**
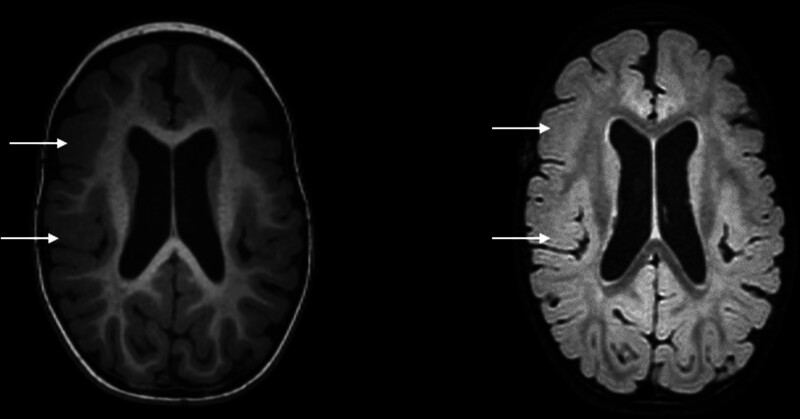
Cranial MRI of the proband (left, T1WI image; r, T2 flair image) revealed reduced medullary volume, fewer gyri, and focal cortical thickening in both hemispheres, indicating MCD. MCD = malformations of cortical development, MRI = magnetic resonance imaging.

**Figure 2.**
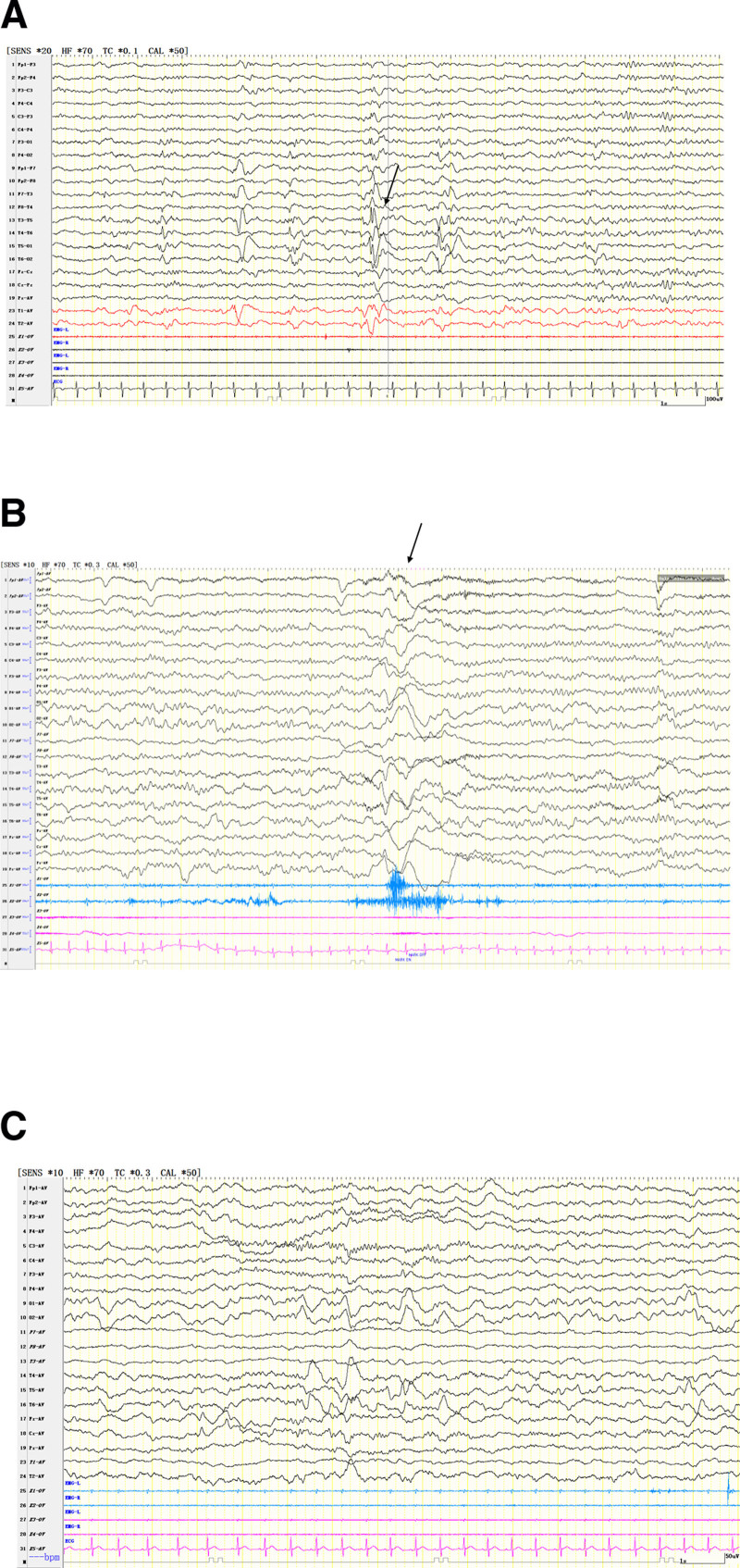
Electroencephalogram. The interictal EEG (A) before the treatment showed low and medium amplitude 1 to 2 Hz spike waves and spike-slow waves in the right parietal region, occipital region, and meso-posterior temporal regions. The ictal EEG (B) showed diffuse low and medium amplitude slow waves episodes, with diamond-shaped muscle electrical bursting, indicating epileptic spasms seizure. The interictal EEG (C) after KD treatment showed 1 to 2 Hz δ activity in bilateral posterior region, with more in the right. EEG = electroencephalogram, KD = ketogenic diet.

Currently, the patient only can sit alone, but cannot crawl, walk, or articulate words such as “dad” or “mom.” A Gesell Developmental Scale assessment revealed moderate developmental delays in adaptive behavior, language, personal-social skills, gross motor skills, and fine motor skills. A comprehensive physical examination only found mild hypotonia in the extremities. Laboratory tests, including blood glucose, plasma ammonia, lactic acid, electrolyte levels, and blood and urine metabolic screening, all yielded normal results. Cranial magnetic resonance imaging demonstrated reduced medullary volume, fewer gyri, and focal cortical thickening in both hemispheres, consistent with MCD (Fig. 1). Electroencephalography (EEG) revealed a large amount of interictal discharges in the right parietal region, occipital region, and meso-posterior temporal regions (Fig. 2A), and monitored epileptic spasms (Fig. 2B) and focal to bilateral tonic-clonic seizures. Given the above evidences, the types of seizures were diagnosed with epileptic spasms and focal to bilateral tonic-clonic seizures. Epilepsy syndrome was diagnosed with infantile epileptic spasms syndrome (IESS).

The patient was initially treated with multiple ASMs, including topiramate, adrenocorticotropic hormone (ACTH), levetiracetam, and sodium valproate; however, his seizures remained refractory. Subsequently, KD treatment was initiated with a fat-to-nonfat ratio of 2:1. Within 1 week of KD initiation, his seizure frequency gradually decreased, and completed seizure control was achieved after 1 month. To date, he has remained seizure-free for 1 year, and follow-up EEG revealed slow waves in the bilateral posterior regions (Fig. 2C). No adverse effects were observed during the KD treatment. Additionally, mild developmental improvements were noted, particularly in language skills.

### 2.2. Genetic analysis

**Figure 3.**
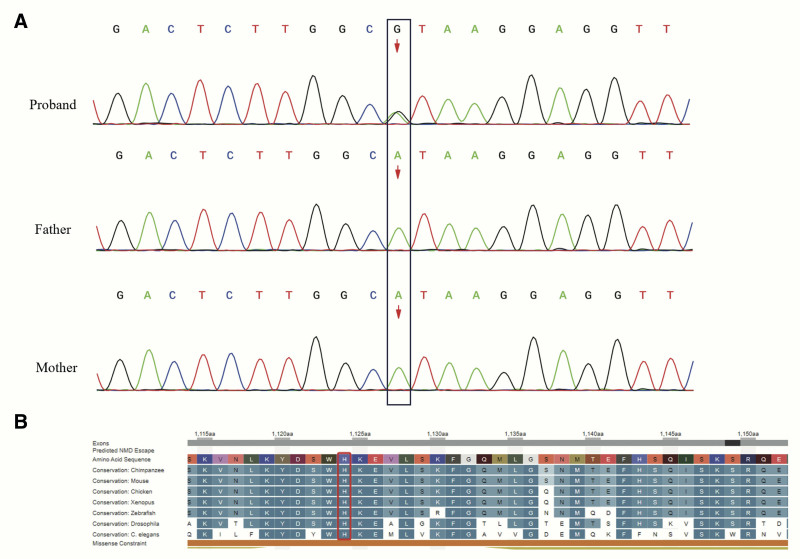
(A) The validation of *DYNC1H1* variants by Sanger sequencing results. *DYNC1H1* gene in both parents were wild-type, indicating a de novo mutation in proband. (B) The conservation assessment of *DYNC1H1* mutation site showed p.His1124 was highly conserved in different species.

Whole-exome sequencing identified a heterozygous variant (c.3371A > G) in exon 14 of the *DYNC1H1* gene. This variant results in the substitution of adenine (A) with guanine (G) at nucleotide position 3371, leading to the replacement of histidine by arginine at amino acid position 1124 (p.His1124Arg). The variant was validated using Sanger sequencing, which included screening both parents. Sanger sequencing confirmed that the *DYNC1H1* gene in both parents was wild-type, indicating that the variant arose as a de novo mutation in the proband (Fig. 3A).

This study was approved by the hospital’s ethics committee, and written informed consent was obtained from the patient’s parents for all diagnostic and treatment procedures. The variant c.3371A > G was identified as a de novo mutation, as Sanger sequencing confirmed that neither parent carried the variant, supporting the criterion PS2_Moderate. The variant was absent from all major public databases, including ExAC, the 1000 Genomes Project, gnomAD, and ESP6500, fulfilling the PM2_supporting criterion. Additionally, other missense variants at the same site resulting in different amino acid substitutions have been previously reported as pathogenic, meeting the PM5 criterion. Given that missense variants in the DYNC1H1 gene are strongly associated with epilepsy and benign variants in this gene are rare, the PP2 criterion was satisfied. Multiple in silico prediction tools consistently indicated that the variant has deleterious effects on the gene or its product, supporting the PP3 criterion. Based on the combined evidence (PS2_Moderate + PM2_supporting + PM5 + PP2 + PP3), this heterozygous variant was classified as “likely pathogenic” according to the American College of Medical Genetics and Genomics (ACMG) guidelines.^[8]^

### 2.3. The conservation assessment of DYNC1H1 gene

In our study, we evaluated the conservation of the variant site using the University of California Santa Cruz Genome Browser (http://genome.ucsc.edu/). The analysis revealed that the histidine residue at position 1124 (p.His1124) is highly conserved across multiple species (Fig. 3B), indicating that substitutions at this site are likely to disrupt protein function.

## 3. Discussion

*DYNC1H1* encodes the heavy chain 1 of the cytoplasmic dynein complex, which plays a critical role in microtubule binding and the recruitment of other dynein components.^[9]^ In this study, we report a case of DEE due to a de novo heterozygous *DYNC1H1* variant c.3371A > G (p.His1124Arg), thereby expanding the genotypic spectrum of *DYNC1H1*-related DEE. Notably, the patient achieved significant seizure control following treatment with KD, suggesting that KD may serve as a potential therapeutic option for patients with *DYNC1H1*-related DEE.

Pathogenic variants in the *DYNC1H1* gene were initially reported in 2010^[10]^ and have since been associated with a broad spectrum of clinical phenotypes. In this study, we reported a de novo missense variant in the *DYNC1H1* gene associated with DEE, characterized by early-onset, drug-resistant epilepsy and neurodevelopmental abnormalities. This finding aligned with previous studies demonstrating that the majority of epilepsy-related *DYNC1H1* variants were de novo missense mutations, which often result in severe epileptic phenotypes accompanied by neurodevelopmental delay or brain malformations.^[1]^ The DYNC1H1 protein consists of several critical functional domains,^[11]^ and epilepsy-related variants are distributed throughout the protein, with a higher concentration observed in the dimerization and stalk domains.^[1]^ In our case, the identified variant (p.His1124Arg) was located within the dimerization domain, and the patient presented with severe DEE and MCD. Mechanistically, previous studies have demonstrated that mutations in the stalk domain impair the interaction between the microtubule-binding domain and microtubules, subsequently interfering with neurodevelopment and contributing to epileptic encephalopathy.^[12]^ However, the specific mechanisms by which variants in the dimerization domain led to epilepsy remain poorly understood and require further investigation.

Epilepsy is a common phenotype associated with *DYNC1H1* gene variants, accounting for nearly 40% of cases in previous studies,^[7]^ with the majority exhibiting drug-resistant epilepsy. In a Chinese cohort of *DYNC1H1*-related epilepsy, over 10% of patients were diagnosed with IESS.^[7]^ Our case was consistent with these findings, as the patient was also diagnosed with IESS and presented with drug-resistant epilepsy. The high incidence of drug-resistant epilepsy in *DYNC1H1*-related cases might be associated with the manifestation of MCD, which are observed in up to 92% of patients with *DYNC1H1*-related epilepsy.^[7]^ The drug-resistant nature of *DYNC1H1*-related epilepsy poses significant therapeutic challenges. In our study, the patient achieved seizure-free following KD treatment after failing to respond to multiple ASMs. To date, therapeutic data on *DYNC1H1*-related epilepsy remain scarce. Only few prior case reports have shown a >50% reduction in seizures after initiating KD in a patient with *DYNC1H1*-related DEE.^[13,14]^ KD is a high-fat, low-carbohydrate, and adequate-protein diet designed to induce ketosis, producing ketone bodies that cross the blood-brain barrier and serve as an alternative energy source for the brain. The precise anti-seizure mechanism of KD in *DYNC1H1*-related DEE remains unclear. We hypothesize that this mechanism might be related to mitochondrial dynamics, as mitochondrial transport is directly or indirectly regulated by the dynein complex and associated proteins involved in retrograde transport, including DYNC1H1.^[15]^ In turn, KD has been shown to influence mitochondrial biogenesis and function.^[16]^

Additionally, we speculate that the role of KD in the treatment of *DYNC1H1*-related DEE was mainly to treat MCD. Growing evidence supports the favorable efficacy and safety of KD in patients with epilepsy and MCD.^[17,18]^ Previous studies suggest that KD exerts anti-seizure effects in structural epilepsy, potentially through anti-inflammatory mechanisms.^[17]^ However, further research is needed to elucidate the underlying mechanisms of KD’s anti-seizure effects in *DYNC1H1*-related epilepsy. Recently, microtubule inhibitors have emerged as potential targeted therapies for *DYNC1H1*-related spectrum disorders.^[19]^ However, their use in *DYNC1H1*-related epilepsy has not yet been explored. Future studies are warranted to evaluate the effectiveness and safety of microtubule inhibitors in this context.

## 4. Conclusion

In this study, we report a de novo heterozygous variant in the *DYNC1H1* gene associated with DEE, which was successfully managed with KD treatment. Our findings expand the genotypic spectrum of *DYNC1H1*-related epilepsy and suggested KD might be an optimized treatment for drug-resistant DEE, providing valuable clinical insights for the management of these patients.

## Acknowledgments

We are very grateful to the child and his parents for their contribution to our study.

## Author contributions

**Resources:** Fen Zhao, Wandong Hu.

**Supervision:** Fen Zhao, Lina Sun, Hongwei Zhang.

**Writing – original draft:** Fen Zhao, Lina Sun.

**Writing – review & editing:** Hongwei Zhang.
